# Pre-Menstrual Dysphoric Disorder (PMDD) in Young People: What We Know About It, the Role of CBT as a Treatment Option, and the Development of High-Quality Psychoeducation Material for Clinical Use

**DOI:** 10.1192/bjo.2022.314

**Published:** 2022-06-20

**Authors:** Judy King, Hannah Wilkinson

**Affiliations:** Berkshire Healthcare NHS Foundation Trust, Berkshire, United Kingdom

## Abstract

**Aims:**

We aim to explain more about PMDD in young people and explore the evidence looking at the role of CBT as an intervention. We aim to develop high-quality psychoeducation material with the involvement of young people, to be able to offer clinically relevant information and empower young people with PMDD.

**Methods:**

A literature search was conducted in 2019 and updated in February 2022 using the Cochrane Library, Psych-info, MEDLINE, Cinahl, EMBASE and Google Scholar, to look at the evidence available for CBT in young people with PMDD or PMS. The search included PMS as well as PMDD due to the heterogeneity in definitions used in studies.

Focus groups with young people are underway to develop high quality written psychoeducation material about PMDD.

**Results:**

There were no specific studies looking at CBT as an intervention in young women under the age of 18 with PMS or PMDD. There was one intervention study with a treatment arm of psychoeducation in 62 young people under 18 with PMS versus a no treatment group (Taghizadeh 2013), with improvement in symptoms after 3 months from baseline reported.

There were more studies available in women over the age of 18. The search identified 3 meta- analyses in 2009 and 2012 (Busse 2009, Lusty, 2009; Kleinstauber 2012) and a more recent systematic review (Landolt 2020). Kleinsteiber et al included 22 RCTs, with a median age of 39, and broadly showed CBT to have a small to medium positive effect size, although any conclusions were limited due to the small numbers involved in the individual trials and methodological flaws. Landolt 2020 looked at CBT or elements of CBT as an intervention arm over the last 30 years or so in woman of all ages. Variations of CBT including virtual, group, couple and psychoeducation alone were included, and all were reported to have favourable outcomes.

**Conclusion:**

There is a huge gap in research looking at PMDD in the adolescent population. Translating research from adults is not ideal. Increasing awareness and developing psychoeducation is a step in the right direction.

